# An estimation of the prevalence of nonmelanoma skin cancer in the U.S.

**DOI:** 10.12688/f1000research.2-107.v1

**Published:** 2013-04-09

**Authors:** Eshini Perera, Rodney Sinclair

**Affiliations:** 1 Department of Dermatology, Epworth Hospital, Richmond, Melbourne, 3121, Australia; 2 Faculty of Medicine, University of Melbourne, Melbourne, 3010, Australia

## Abstract

Nonmelanoma skin cancer (NMSC) is the most commonly diagnosed cancer in Australia and has a significant impact on the cost of and use of healthcare resources. Current estimates of NMSC in the USA are 3.5 million cases in 2010 compared to 1.63 million cases of all other cancers combined.  However, we believe that this figure significantly underestimates the prevalence of NMSC in the USA.  We calculated that melanoma is diagnosed 5.7 times more in the USA than in Australia.  In Australia, in 2010, there were 767,000 NMSC diagnoses.  If the ratio of melanoma: NMSC is constant in both Australia and the USA, then there should be 5.7 times the number of NMSC in the USA or 4.3 million cases.  The assumptions that underlie this calculation are discussed.

## Introduction

Nonmelanoma skin cancer (NMSC) includes basal cell carcinoma and squamous cell carcinoma of the skin, and is common in both Australia and the USA. In fact, in both Australia and the USA, NMSC is more common than all other forms of cancer combined ^1– 3^. NMSCs are predominately managed privately in an outpatient setting. NMSC is not a reportable cancer and NMSC data are not recorded on national cancer registries in either Australia or the USA.

Estimates of the incidence of NMSC in both Australia and the USA are obtained by periodic surveys. Incidence data record an individual’s first episode of skin cancer within a calendar year. As NMSC patients commonly develop multiple primary cancers, incidence data are likely to significantly underestimate the burden of disease.

In order to calculate NMSC prevalence in Australia, data from more than 8 million skin cancers treated between 1 January 1997 and 31 December 2010 ^4^ were evaluated by Fransen *et al.* Data from the study were obtained from Medicare Australia. The population of Australia grew 23% from 18.3 million in 1997 to 22.6 million in 2010 ^5^. Roughly 95% of the population or 21,470000 people are of European or Anglo-Saxon descent. Medicare Australia is a Commonwealth Government-funded universal health insurance scheme. Medicare Australia data captures 98% of all skin cancer treatments in Australia. Fewer than 2% of NMSC lesions are treated in public hospitals that record data independently ^6^. All skin cancer treatments in Australia, except those provided in a public hospital, are individually itemized and recorded by Medicare Australia for the purpose of patient reimbursement. Item numbers for the excision, cryotherapy, curettage or laser treatment of skin cancers require histological confirmation of the diagnosis or clinical diagnosis by a registered dermatologist prior to Medicare coding.

The total number of NMSC treatments increased by 186% from 412,493 in 1997 to 767,347 in 2010 ^4^. The number of treatments are estimated to increase to 938,991 (95% CI, 901,047–976,934) by 2015 ^4^.

So, what is the prevalence of NMSC in the USA? Rogers *et al.* estimated that in 2010 more than 3.5 million skin cancers were diagnosed in over 2 million people ^2^. The number of practicing dermatologists in USA in 2010 was 9598 ^7^. This figure suggests on average that each dermatologist treats 364 NMSCs a year, or one a day. Anecdotally, this figure seems low, even accounting for differences of skin cancer prevalence in each state.

Another way to estimate the burden of NMSC in USA is to compare the incidence of melanoma in the USA to that in Australia. Melanoma is a cancer that is reportable in both the USA and Australia. Reporting is mandatory and data on melanoma prevalence are likely to be more complete compared to data on NMSC.

In 2010, it was predicted that 68,130 melanomas would be recorded on the cancer registry in the USA ^3^ and 11,900 were predicted for the national cancer registry in Australia in 2010 ^1^. This amounts to 7.1 melanomas per dermatologist per year in the USA and 21.6 melanomas per dermatologist in Australia in 2010.

National registries data indicate that 5.7 times the number of melanomas were diagnosed in the USA among a population that is 14 times larger than Australia. One model ^6^ assumes that the climate, ethnicity, sun exposure behaviour and population differences that lead to the higher incidence of melanoma in Australia do not affect the ratio of melanoma to NMSC. We also assume that melanoma is recorded with equal accuracy and completeness in both the USA and Australia. Based on these assumptions, the number of NMSCs treated in the USA in 2010 would also be 5.7 times the number of NMSCs treated in Australia. As 767,000 NMSCs were treated in Australia in 2010, we estimate that 4.3 million NMSC were treated in USA.

The population of the USA was 308.7 million in 2010 and 72.4% were White or European American ^8^, 12.6% were black or African American ^8^. The remaining 15% comprised people of different ethnicities including Hispanic or Latino, Asian, Native American, Hawaiian and Pacific Islanders ^8^. The skin cancer with the least ethnic variation in incidence is acral lentiginous melanoma (ALM). ALM only represents 2–3% of all melanoma diagnosed in the USA ^9^. If ALM were excluded from the total number of melanomas diagnosed in the USA in 2010, that reduces our estimate of NMSC in USA from 4.3 to 4.21 million cases.

This is roughly an extra 700,000 cases on top of the current estimations of NMSC by Rogers *et al* ^2^. This now equates to approximately 1.2 NMSCs per US dermatologist per day. Anecdotal experience would suggest this figure still underestimates the true burden of NMSC in USA. A prospective registry may be required to better estimate the full burden of NMSC and to ensure that there are adequate physical resources and manpower to treat NMSC patients in the USA.

## References

[1] Australian Institute of Health and Welfare & Australasian Association of Cancer Registries. Cancer in Australia: an overview 2010. (Cancer series no 60). AIHW Cat. No 56. Canberra: Australian Institute of Health and Welfare;2010 Reference Source

[2] Rogers HW Weinstock MA Harris AR: Incidence Estimate of Nonmelanoma Skin Cancer in the United States, 2006. *Arch Dermatol.* 2010;146(3):283–287 10.1001/archdermatol.2010.19 20231499

[3] American Cancer Society. Cancer facts and figures 2010. Atlanta: American Cancer Society;2010 Reference Source

[4] Fansen M Karahalios A Sharma N: Non-melanoma skin cancer in Australia. *Med J Aust.* 2012;197(10):565–568 10.5694/mja12.10654 23163687

[5] Australian Bureau of Statistics. Population projections, Australia, 2006 to 2101. (Cat. No. 3222.0). Canberra: Australian Bureau of Statistics;2008 Reference Source

[6] Sinclair R: Nonmelanoma skin cancer in Australia. *Br J Dermatol.* 2013;168(1):1–2 10.1111/bjd.12167 23278555

[7] Yoo JY Rigel DS: Trends in Dermatology: Geographic Density of US Dermatologists. *Arch Dermatol.* 2010;146(7):779 10.1001/archdermatol.2010.127 20644040

[8] Humes K Jones N Ramirez R: Overview of Race and Hispanic Origin: 2010. Washington DC. U.S. Census Bureau;2011 Reference Source

[9] Bradford PT Goldstein AM McMaster ML: Acral lentiginous melanoma: incidence and survival patterns in the United States, 1986–2005. *Arch Dermatol.* 2009;145(4):427–34 10.1001/archdermatol.2008.609 19380664 PMC2735055

