# Supplementary material for: An estimation of the prevalence of nonmelanoma skin cancer in the U.S
Source: F1000Res. 2013 Apr 9;2:107. [Version 1] doi: 10.12688/f1000research.2-107.v1 (PMC3752668; doi:10.12688/f1000research.2-107.v1)
Supplement: Peer review history

# Peer review: Referee response for version 1

**Published:** 2013-04-15

**Authors:** Kamran Ghoreschi

**Affiliations:** Department of Dermatology, Eberhard Karls University of Tübingen, Tübingen, Germany

The findings of this commentary are based on the estimations and calculations made by the authors of the work. The commentary itself is interesting, and helps provoke the idea that there is a higher prevalence of non-melanoma skin cancer (NMSC) in the US. However, the authors quite rightly demonstrate the potential weaknesses in the epidemiology of the disease; it is still difficult to collect data on the epidemiology of skin cancer even in Western countries. The authors do encourage prospective registries to help estimate NMSC, however as discussed, sun exposure and other factors have to be considered too in order to provide a more definitive answer for the prevalence of NMSC in the US.

I have read this submission. I believe that I have an appropriate level of expertise to confirm that it is of an acceptable scientific standard.

# Peer review: Referee response for version 1

**Published:** 2013-04-15

**Authors:** Gabriella Fabbrocini

**Affiliations:** Department of Dermatology, University of Naples Federico II, Naples, Italy

This study can be considered very interesting for the new epidemiological insights that it provides. The study is interesting for the evaluation of relationship between melanoma and non-melanoma skin cancers (NMSC) in Australia and in USA; it highlights the underestimation of NMSC prevalence in USA, so it opens new inputs for a better demographic evaluation of these tumors.

The title is simple and appropriate for the content of the article and the abstract can be considered a good summary of the work. The design of the study, and relative materials and methods are clearly explained and appropriate for this study. All data are clearly specified, clearly exposed and well schematized. The conclusions respect the basis of the study results.

I have read this submission. I believe that I have an appropriate level of expertise to confirm that it is of an acceptable scientific standard.
